# Anti-Inflammatory and Anti-Acne Effects of *Hamamelis virginiana* Bark in Human Keratinocytes

**DOI:** 10.3390/antiox11061119

**Published:** 2022-06-05

**Authors:** Stefano Piazza, Giulia Martinelli, Urska Vrhovsek, Domenico Masuero, Marco Fumagalli, Andrea Magnavacca, Carola Pozzoli, Luisa Canilli, Massimo Terno, Marco Angarano, Mario Dell’Agli, Enrico Sangiovanni

**Affiliations:** 1 Department of Pharmacological and Biomolecular Sciences, University of Milan, 20133 Milan, Italy; stefano.piazza@unimi.it (S.P.); giulia.martinelli@unimi.it (G.M.); marco.fumagalli3@unimi.it (M.F.); andrea.magnavacca@unimi.it (A.M.); carola.pozzoli@unimi.it (C.P.); marco.angarano@guest.unimi.it (M.A.); 2 Department of Food Quality and Nutrition, Research and Innovation Centre, Fondazione Edmund Mach (FEM), 38098 S. Michele all’Adige, Italy; urska.vrhovsek@fmach.it (U.V.); domenico.masuero@fmach.it (D.M.); 3 Institute Ganassini S.p.A. (Istituto Ganassini), 20139 Milan, Italy; l.canilli@ganassini.it (L.C.); dir.tecnica@ganassini.it (M.T.)

**Keywords:** *Hamamelis virginiana*, acne, proanthocyanidins, hamamelitannin, skin inflammation, keratinocytes, *Cutibacterium acnes*

## Abstract

*Cutibacterium acnes* (*C. acnes*) is recognized as one of the main triggers of the cutaneous inflammatory response in acne vulgaris, a chronic skin disorder with a multifactorial origin. Witch hazel (*Hamamelis virginiana* L.) is a plant widely used for skin inflammatory conditions, with some preliminary anti-inflammatory evidence on the skin, but lacking data on acne conditions. This study aimed to evaluate the effect of a glycolic extract from *Hamamelis virginiana* bark (HVE) versus *C. acnes*-induced inflammation in human keratinocytes (HaCaT). Phytochemical investigations of HVE identified hamamelitannin (HT) and proanthocyanidins as the most abundant compounds (respectively, 0.29% and 0.30% *w*/*w*_extract_). HVE inhibited *C. acnes*-induced IL-6 release (IC_50_: 136.90 μg/mL), by partially impairing NF-κB activation; however, no antibacterial or antibiofilm activities were found. In addition, HVE showed greater anti-inflammatory activity when TNF-α was used as a proinflammatory stimulus (IC_50_ of 38.93 μg/mL for IL-8 release), partially acting by antioxidant mechanisms, as shown for VEGF inhibition. The effects of HVE are primarily based on the proanthocyanidin content, as HT was found inactive on all the parameters tested. These results suggest further investigations of HVE in other inflammatory-based skin diseases.

## 1. Introduction

Acne vulgaris is an inflammatory-based condition that affects almost 80% of young people during puberty and represents one of the top three prevalent skin inflammatory conditions [1]. The pathogenesis of acne is multifactorial and includes androgen stimulation of sebaceous glands, *Cutibacterium acnes* (*C. acnes*) colonization, and inflammation. The proliferation of *C. acnes* triggers the release of proinflammatory cytokines at cutaneous levels, such as interleukin-1β (IL-1β), IL-8, granulocyte-macrophage colony-stimulating factor (GM–CSF), and tumor necrosis factor α (TNF-α), through the activation of nuclear factor kappa B (NF-κB) [2].

There is no election treatment for acne, and chronic use of antibiotics during acne promotes the generation of antibiotic-resistant strains, also exacerbating adverse effects [3]. With these premises, the discovery of alternative or multitarget approaches seems fundamental.

Phytotherapy of acne vulgaris has the potential benefit of acting through different mechanisms, by inducing antibacterial, anti-inflammatory, and antioxidant activities. Among the different natural products, plants rich in tannins are potentially useful in acne, also thanks to their astringent activity [4].

*Hamamelis virginiana* L. (or witch hazel) is a deciduous tree native to the Atlantic coast of North America, widely included in skin care products; however, scientific evidence regarding its efficacy in acne is scarce.

Witch hazel has a long history of medicinal use. The Indigenous Native American populations employed leaves and bark as remedies for a wide list of conditions, which covered (but were not limited to) fevers, colds, and skin inflammations [5]. The European Medicines Agency (EMA) reported on the consolidated use of hamamelis hydroalcoholic leaf or bark extracts (ethanol 30–60%) for circulatory and skin disorders [6,7] and the distillate from fresh or dried parts (ethanol 6–15%) for ocular discomforts [8]. The German Commission E (updated 2012) monographs confirmed the use of witch hazel for minor injuries of the skin, primarily based on human experience and long-standing use [9].

The most abundant polyphenols of *H. virginiana* leaves and bark are hydrolysable and condensed tannins (proanthocyanidins). According to the EMA Committee on Herbal Medicinal Products (HMPC), hamamelis bark contains a higher percentage of tannins than leaves (8–12% vs. 3–10%) and a higher content of hamamelitannin (HT, 1–7%), the main characteristic constituent of witch hazel; but tannins are almost absent in distillates prepared from the same plant materials [10].

Despite the wide traditional use of witch hazel and the presence of some clinical studies [11,12,13,14,15], the mechanisms of the anti-inflammatory actions of hamamelis extracts on the skin are poorly documented. Human studies reported in the literature used plant distillates, which showed conflicting efficacy in dermatitis. On the other hand, a pilot study involving seven healthy volunteers tested the efficacy of a semi-solid formulation (containing 1% witch hazel procyanidins) on sodium lauryl sulfate-induced skin inflammation [16]. The topical anti-inflammatory effects of proanthocyanidins from hamamelis bark were also demonstrated in the croton oil ear edema test in mice [17].

Some in vitro studies attributed the superior inhibitory effects of proanthocyanidins on arachidonate 5-lipoxygenase (5-LOX) and platelet-activating factor (PAF) compared to HT [18], which, mainly showed antioxidant activities [19] and protective effects on endothelial toxicity induced by TNF-α [20]. Two preliminary studies on human cultured keratinocytes suggested the possible role of gallotannins and proanthocyanidins in the antioxidant and cytoprotective effects of hamamelis bark extract [16,21]. In addition, witch hazel extract showed antibacterial activity in vitro [22,23].

Based on these premises, the aim of this work was to evaluate the potential anti-acne activity of *H. virginiana* bark, by assessing the antioxidant, antibacterial, and anti-inflammatory activities in human keratinocytes.

The results of this study show that witch hazel acts mainly on inflammation, thanks to the number of condensed tannins; however, it did not show inhibitory activity on *C. acnes*.

## 2. Materials and Methods

The glycolic extract from the twigs and bark of *H. virginiana* L. (HVE) was provided by Istituto Ganassini (via Carlo Boncompagni, Milan, Italy). HT, (−)-epigallocatechin (EGC), (−)-epicatechin gallate (ECG), (−)-epigallocatechin gallate (EGCG), and (−)-epicatechin (EC) were purchased from PhytoLab GmbH & Co. KG (Vestenbergsgreuth, Germany), and dissolved with DMSO at a final concentration of 20 mM. The concentration of 20 mM of HT corresponds to 10 mg/mL.

### 2.1. Phytochemical Characterization

Phenolic compounds were determined according to Vrhovsek et al. (2012) [24]. The glycolic extract (447 mg) was dissolved in 5 mL of methanol.

HT was determined using the chromatographic method reported by Gasperotti et al. (2010) [25]. The standard of HT was added to the method and a calibration curve was performed with the relative standard of HT.

### 2.2. Analysis of Condensed Tannins

Condensed tannins were determined according to Arapitsas et al. (2021) [26].

### 2.3. Folin–Ciocalteu Assay

To assess the total phenolic content (TPC), HVE was dissolved in 800 μL of water (dilution 1:500); then, 50 μL of 2 N Folin–Ciocalteu reagent (Merck Life Science, Milano, Italy) and 150 μL of 20% (*w*/*v*) Na_2_CO_3_ were added. After 30 min of incubation at 37 °C, samples were transferred to plastic cuvettes, and absorbance was measured with a Jasco V630 Spectrophotometer at a wavelength of 765 nm against a gallic acid calibration curve.

### 2.4. ORAC Test

The oxygen radical absorbance capacity (ORAC) assay was carried out according to Nwakiban A.P.A. et al. [27]. Briefly, an aliquot from stock solutions of HT (25 mM) and HVE (250 mg/mL) was distributed into a black 96-well plate and diluted to a volume of 20 μL. Then, 120 μL of fluorescein solution (70 nM final concentration), previously prepared with a phosphate buffer (pH 7.4, 75 mM), was added to each well. Peroxyl radicals were generated by adding 60 μL of AAPH 40 mM (Merck Life Science, Milan, Italy). The plate was put in a multiplate reader (Victor X3, PerkinElmer, Waltham, MA, 02451, USA) and the fluorescence detector was set at excitation and emission wavelengths of 484 and 528 nm, respectively. The fluorescence was read, after shaking, every 2 min for 60 min at 37 °C. Trolox (0–120 μM) was used as a reference inhibitor. The area under the curve (AUC) of each extract was calculated and the results were expressed as mmol Trolox equivalent.

### 2.5. DPPH Test

DPPH test was performed according to Fracassetti D. et al. [28]. The DPPH solution was diluted with methanol to obtain 1.00 ± 0.03 absorbance units at 515 nm. In a 96-well microplate (Euroclone, Pero, Italy), 245 μL of DPPH solution was placed in each well and 5 μL of the sample was added. The sample was dissolved in 70% methanol; after centrifugation, it was serially diluted. After 50 min at rt, each sample was measured by a microplate reader (Envision, Perkin Elmer, Waltham, MA, 02451, USA). A calibration curve was made by adding an increasing concentration of Trolox ranging from 0 to 2.5 mmol. Each concentration was assayed in triplicate, as well as each sample. Results were expressed as μmol Trolox equivalents per g of powder.

### 2.6. Cell Culture

HaCaT cells (CVCL-0038; Cell Line Service, Eppelheim, Germany) are spontaneously immortalized human keratinocyte cell lines from adult skin. Cells were grown in Dulbecco’s Modified Eagle culture Medium (DMEM; Merck Life Science, Milano, Italy) supplemented with 10% heat-inactivated fetal bovine serum (Euroclone, Pero, Italy), 100 U/mL penicillin, 100 μg/mL streptomycin (Pen Strep Gibco^TM^; Thermo Fisher Scientific, Monza, Italy), and 2 mM L-glutamine (Thermo Fisher Scientific, Monza, Italy). Cells were incubated at 37 °C in a humidified atmosphere containing 5% CO_2_. Every 4 days, cells were detached from 75 cm^2^ flasks (Primo^®^; Euroclone, Pero, Italy) using Trypsin-EDTA 0.25% (Gibco^TM^; Thermo Fisher Scientific, Monza, Italy), counted, and sub-cultured in a new flask or seeded in 24-well plates (Falcon^®^; Corning Life Sciences, Amsterdam, The Netherlands) for the biological tests.

### 2.7. Cutibacterium Acnes Culture and Infection

A sequenced strain of *Cutibacterium acnes* (*C. acnes*), sampled from facial acne (ATCC^®^ 6919TM, LGC standards s.r.l., Milano, Italy), was grown by solid culture and used to infect HaCaT cells. The bacteria were stored at −80 °C, at the optical density (O.D.) of 5, in cryovials containing reinforced clostridial medium 30% (Oxoid, Hampshire, UK), glycerol 20%, and defibrinated sheep blood 50% (TCS Biosciences Ltd., Oxoid, Hampshire, UK). The solid culture was achieved using agar-blood Petri dishes, containing RCM, agar 1% (Merck Life Science, Milan, Italy), and defibrinated sheep blood 5%. Petri dishes were inoculated with 100 μL of the store aliquots of bacteria, sealed in plastic bags containing an anaerobic atmosphere generated by CO_2_ GasPak^TM^ EZ (Becton Dickinson, Sparks, MD, 21152 USA), and then placed in a cell incubator (37 °C) for 48 h before HaCaT cell infection. At the time of infection, bacteria were collected and suspended in PBS 1X solution, counted by the optical method at 600 nm in 1 mL cuvette (Biochrom Libra S22 UV/VIS spectrophotometer), and directly diluted in the medium of cultured HaCaT cells, with a final O.D. of 0.1.

### 2.8. Measurement of the Antibacterial Activity

The activity of HVE or HT against the growth of *C. acnes* was evaluated by the serial dilution method. Briefly, HVE (125–1000 μg/mL) or HT (3–25 μg/mL) were diluted in RCM and placed in a 96-U-bottom well plate (Greiner Bio-one, Milan, Italy) with a final volume of 100 μL. Then, 100 μL of RCM containing the bacterial suspension (final O.D. = 0.1) was added to the samples. The plate was incubated for 24 h under anaerobic conditions to allow the proliferation of *C. acnes*, then the optical density at 600 nm was read. The formation of biofilm was measured following the same procedure of treatment, after which planktonic cells were removed and adherent cells were stained with crystal violet (Remel Europe Ltd., Dartford, United Kingdom) (CV) 1% in distilled water. Then, the excess CV was removed by washing 3 times with distilled water. Stained cells were extracted with 33% acetic acid and the absorbance of the resulting solution was measured at 600 nm. The results were expressed as % relative to the control bacterial growth. The antibiotic erythromycin (0.02 μg/mL) was used as a reference inhibitor.

### 2.9. Cell Viability

The HaCaT cell morphology before and after treatment was assessed by light microscope inspection. Cell viability was measured after the treatments (24 h) by the 3,4,5-dimethylthiazol-2-yl-2-5-diphenyl tetrazolium bromide (MTT) method. This method measures the activity of the succinate dehydrogenase at the mitochondrial level, which is an index of cell viability. No cytotoxicity was observed after the treatment with HVE (0–250 μg/mL) or HT (4.8 μg/mL, corresponding to 10 μM) (Figure S1).

### 2.10. HaCaT Cell Treatment

HaCaT cells were cultivated in 24-well plates (6 × 10^4^ cells/well) for 48 h, to reach the cell confluence. Then, cells were challenged by TNF-α (10 ng/mL) or *C. acnes* infection (O.D. = 0.1) for 6 or 24 h, according to the peak of stimulation of each inflammatory marker, as reported in the results section. HVE (0–250 μg/mL) or HT (0–5 μg/mL) were treated along with the different pro-inflammatory stimuli. Epigallocatechin gallate (20 μM) and apigenin (20 μM) were used as well-known anti-inflammatory polyphenols, against TNF-α- and *C. acnes*-induced inflammation, respectively. The reference compound for the inhibition of VEGF release was apigenin (20 μM).

For the measurement of intracellular ROS cells were cultivated in 96-well black plates (6 × 10^4^ cells/well) for 48 h, to reach the cell confluence. Then, cells were pre-treated for 24 h with HVE (250 μg/mL) or HT (10 μg/mL). Trolox (500 μM) and apigenin (20 μM) were used as antioxidant reference compounds.

For the assessment of catalase (CAT) activity, cells were cultivated in 6-well plates (10^5^ cells/well) for 48 h. Then, cells were treated for 24 h with HVE (250 μg/mL) or HT (10 μg/mL) along with H_2_O_2_ (500 μM).

### 2.11. Measurement of Intracellular ROS

At the end of the 24 h pretreatment, cells were washed with warm PBS and incubated at 37 °C with 25 μM CM-H2DCFDA (Invitrogen^TM^, Thermo Fisher Scientific, Monza, Italy) in HBSS without phenol red (Merck Life Science, Milano, Italy). After 30 min, cells were washed twice with PBS and treated with 1 mM H_2_O_2_ in serum-free medium, except for the control to which only medium was added. Following 1 h of incubation at 37 °C, cells were washed with PBS; 100 μL of PBS was added to each well, and fluorescence was read using a multiplate reader (Victor ×3; PerkinElmer, Milano, Italy) at ex. 490 nm/em 535 nm. The results were normalized for the protein content determined by the sulforhodamine B (Merck Life Science, Milano, Italy) assay, according to Ng and Ooi [29].

### 2.12. Measurement of CAT Activity

For the assessment of CAT activity, HaCaT cells were detached using a cell scraper at 24 h treatment and homogenized in cold potassium phosphate buffer (K_3_PO_4_ 50 mM, EDTA 1 mM, pH 7.0); then, cell lysates were analyzed for total protein content (BCA method, Euroclone, Pero, Italy). The CAT activity of each sample (20 μL) was measured by a colorimetric assay, according to the manufacturer (Catalase assay kit, Cayman chemical, Ann Arbor, MI, USA). In brief, the capacity of each sample to oxidize methanol into formaldehyde (CAT peroxidatic activity) was determined by the colorimetric reaction of the latter with 4-amin-3-hydrazino-5-mercapto-1,2,3-triazole (Purpald), leading to a purple adduct with absorbance at 535 nm (EnVision; PerkinElmer, Milano, Italy). Absorbance data were normalized on total protein content and expressed as CAT activity on μg of sample proteins (mean ± SEM).

### 2.13. Measurement of the Release of Inflammatory Mediators: IL-8 and VEGF-A

The release of IL-8 and VEGF-A by HaCaT cells was evaluated by enzyme-linked immunosorbent assay (ELISA) on culture media. Human IL-8 and VEGF-A ELISA assays (PeproTech, London, UK) were conducted following the instructions from the manufacturers. In brief, IL-8 and VEGF-A measurements were based on the sandwich ELISA method: Corning 96-well EIA/RIA plates (Merck Life Science, Milano, Italy) were coated overnight at room temperature with the capture antibody; then, after the interaction of samples (cell medium or curve standard protein) with a biotinylated secondary antibody, the amount of target protein was detected by measuring the absorbance resulting from the colorimetric reaction between the horseradish peroxidase enzyme and 2,2-azino-bis(3-ethylbenzothiazoline-6-sulfonic acid) (ABTS) (Merck Life Science, Milano, Italy). The signal from the sample was acquired using a multiplate reader (Victor X3; PerkinElmer, Milano, Italy) at 405 nm and compared with the calibration curve. The results (mean ± SEM of at least three experiments) were expressed as a percentage relative to the stimulated control, which was arbitrarily assigned the value of 100%.

### 2.14. Measurement of NO Release

The release of NO was measured by the Griess reagent (40 mg/mL) (Merck Life Science, Milano, Italy), added with a 1:1 ratio to the sample medium (100 μL) from HaCaT cells in a clear 96-well plate. NaNO_2_ was used as a reference standard to perform a calibration curve (0–10 μg/mL). After 5 min at rt, samples were read at 550 nm using a multiplate reader (Victor ×3; PerkinElmer, Milano, Italy). The results (mean ± SEM of at least three experiments) were expressed as a percentage relative to the stimulated control, which was arbitrarily assigned the value of 100%.

### 2.15. Measurement of the NF-κB-Driven Transcription

Before transfection, the medium of HaCaT cells was replaced with a serum-depleted culture medium. Then, cells were transiently transfected with a reporter NF-κB-Luc plasmid (250 ng per well), containing the luciferase gene under the control of the E-selectin promoter characterized by three κB responsive elements, using Lipofectamine^®^ 3000 Transfection Reagent (Invitrogen^®^; Thermo Fisher Scientific, Monza, Italy). The plasmid NF-κB-Luc was a gift from Dr. N. Marx (Department of Internal Medicine-Cardiology, University of Ulm; Ulm, Germany). The day after (16 h from transfection), cells were treated with *C. acnes* or TNF-α as previously stated. At the end of the treatment, the amount of luciferase produced in the cells was assessed using Britelite^TM^ Plus reagent (Perkin Elmer, Milano, Italy) according to the manufacturer’s instructions. The luminescence derived from the reaction between luciferase and luciferin was measured with a multiplate reader (Victor ×3; PerkinElmer, Milano, Italy). The results (mean ± SEM of at least three experiments) were expressed as a percentage relative to the stimulated control, which was arbitrarily assigned the value of 100%.

### 2.16. Statistical Analysis

All data were expressed as the mean ± SEM of at least three independent experiments. ELISA assays were analyzed by unpaired one-way analysis of variance (ANOVA), followed by the Bonferroni post hoc test. Statistical analyses were performed using GraphPad Prism 8.0 software (GraphPad Software Inc., San Diego, CA, USA). Values of *p* < 0.05 were considered statistically significant.

## 3. Results

### 3.1. Phytochemical Analysis of HVE

The first investigation of HVE aimed to characterize its phytochemical profile. The TPC, measured through the Folin–Ciocalteu assay, was 6.17 ± 0.33 mg of gallic acid equivalents (GA eq.) for 1 g of extract, corresponding to 0.62% (w. GA eq./w. extract). According to the HMPC’s monographs (EMA/HMPC/114585/2008), *Hamamelis virginiana* bark contains a huge amount of condensed tannins, flavonols, and gallotannins, such as HT. Consequently, the content was verified by an MS analysis, which confirmed a noteworthy amount of HT (0.29% *w*/*w* (extract)) and condensed tannins (0.30%) (Table 1), accounting for most of the previously measured TPC.

Accordingly, the presence of representative flavonoids, phenolic acids, and procyanidin dimers was negligible. The chemical nature of condensed tannins was ascribed to polymeric galloylated proanthocyanidins, with a high average degree of polymerization (aDP = 19). The main monomeric unit identified by phloroglucinolysis was epicatechin gallate, followed by epigallocatechin, catechin, and epicatechin.

### 3.2. HVE Inhibits Intracellular ROS Production

Few of the previous studies attributed a cytoprotective effect to *Hamamelis virginiana* L. bark extract against skin oxidative stress [21]. As a first step for the characterization of the biological properties of HVE, we evaluated the radical scavenging activities of HVE and HT in two cell-free systems, ORAC (Table 2) and DPPH (Table 3) assays. Considering that HT corresponds to 0.3% *w*/*w* of HVE, the scavenging power of HT contributes only partially to the effect of HVE.

**Figure 1:**
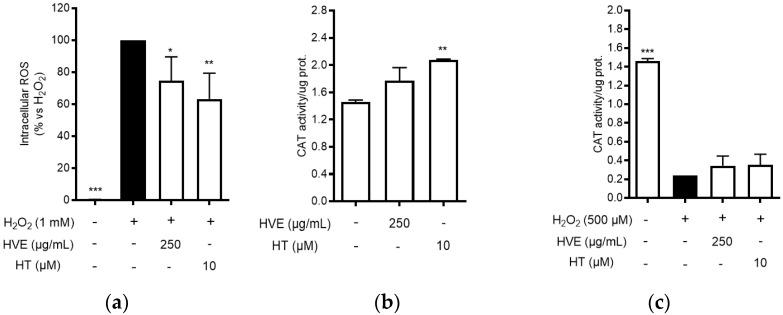
Effect of HVE (250 μg/mL) and HT (10 μM) on oxidative stress induced by H_2_O_2_ in HaCaT cells. (**a**) Effect of HVE and HT pre-treatment (24 h) on intracellular ROS levels after 1 h of stimulation with H_2_O_2_ (1 mM). Trolox (500 μM) and apigenin (20 μM), representing reference antioxidant compounds, inhibited ROS production by −49% and −37%, respectively. (**b**) Effect of HVE and HT treatment along with H_2_O_2_ (500 μM) (24 h) on catalase activity; * *p* < 0.05, ** *p* < 0.01, *** *p* < 0.001 vs. stimulus. (**c**) Effect of HVE and HT on basal CAT activity; ** *p* < 0.01, vs. CTRL.

Consequently, the antioxidant activity of HVE and HT was further characterized by measuring intracellular ROS production and CAT activity in cell-based assays. HaCaT cells were treated for 24 h with HVE (250 μg/mL) or HT (10 μM) before inducing oxidative stress by H_2_O_2_ for 1 h. Both HVE and HT significantly inhibited the intracellular ROS production in comparison to H_2_O_2_ (Figure 1a), without altering basal levels of ROS (data not shown). The data correlated with a slight enhancement of CAT activity after 24 h of treatment in the absence of a pro-oxidant stimulus (significant for HT only) (Figure 1b). On the contrary, HVE and HT were unable to counteract the depletion of CAT activity induced by H_2_O_2_ (24 h), although a slight but not significant recovery (two-fold) was observed in comparison to the oxidative condition (Figure 1c).

These preliminary data suggested that HVE may preferentially exhibit a preventive effect on oxidative stress, probably through direct radical scavenger activity and by enhancing antioxidant defense. Of note, in line with the ORAC and DPPH assays, the bioactivity of HT was observed at concentrations 10-fold higher than those present in the extract, thus supposing a major relevance for polymeric proanthocyanidins.

### 3.3. HVE Inhibits C. acnes-Induced Inflammation in HaCaT Cells

**Figure 2:**
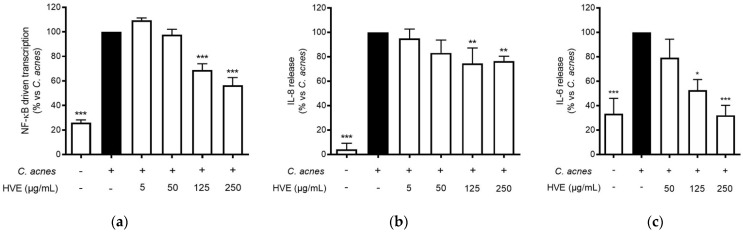
Effect of HVE treatment (5–250 μg/mL) on the NF-κB-driven transcription (**a**) for 6 h, IL-8 release (**b**), and IL-6 release (**c**) in HaCaT cells infected with *C. acnes* (O.D. = 0.1) for 24 h. Apigenin (20 μM), representing the reference inhibitor, abrogated the NF-κB-driven transcription (−100%) and inhibited the release of IL-8 (−42%) and IL-6 (−97%). The amount of IL-8 in the stimulated condition was 426.41 ± 96.97 pg/mL. * *p* < 0.05, ** *p* < 0.01, *** *p* < 0.001 vs. stimulus.

Tannin-rich extracts from *Hamamelis virginiana* L. are traditionally used to treat various skin infections due to their putative antioxidant, anti-inflammatory, and antibacterial properties [18,22]. The release of IL-8 from keratinocytes, resulting from the interaction between *C. acnes* and TLR-2 on the cellular membrane, has been recognized to play a fundamental role in the pathogenesis of inflammatory acne [30,31]. The most important regulator of this event at the transcriptional level is NF-κB, which enhances the antibacterial response, but also the exacerbation of the inflammatory outcomes [32]. In our experiments, HVE inhibited the NF-κB-driven transcription in a concentration-dependent manner (IC_50_ = 136.90 ± 17.22 μg/mL) (Figure 2a) and partially counteracted IL-8 release (−26.79%) at 250 μg/mL (Figure 2b). HVE completely inhibited IL-6 release induced by *C. acnes* at the maximum concentration tested of 250 μg/mL (IC_50_ = 136.90 ± 17.22 μg/mL) (Figure 2c).

Since HT was the main compound identified in HVE, the same experiments were conducted using HT in a concentration range comparable to that present in the extract (0.25–2.5 μg/mL of HT, corresponding to 0.5–5 μM). Surprisingly, HT was devoid of inhibitory activity at the concentrations tested in all these parameters (Figure S2).

### 3.4. The Bioactivity of HVE Is Independent of the Antibacterial Effect on C. acnes

**Figure 3:**
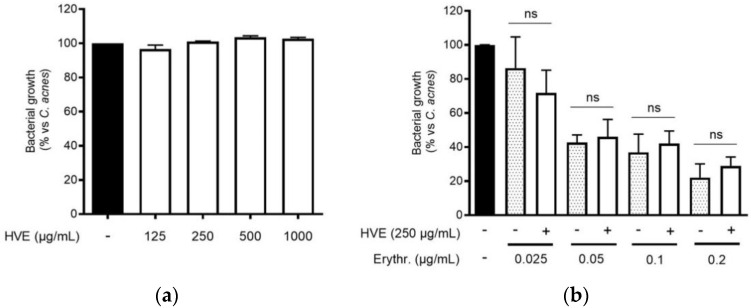
Effect of HVE treatment (125–1000 μg/mL) on the growth of *C. acnes* (O.D. = 0.1) for 24 h (**a**). Erythromycin (0.2 μg/mL) was used as the antibiotic control (−80% of growth vs. control). Evaluation of the co-administration of HVE (250 μg/mL) and erythromycin (0.025–0.2 μg/mL) on bacterial growth (**b**). HVE + erythromycin not significant (ns) vs. equivalent concentration of erythromycin.

**Figure 4:**
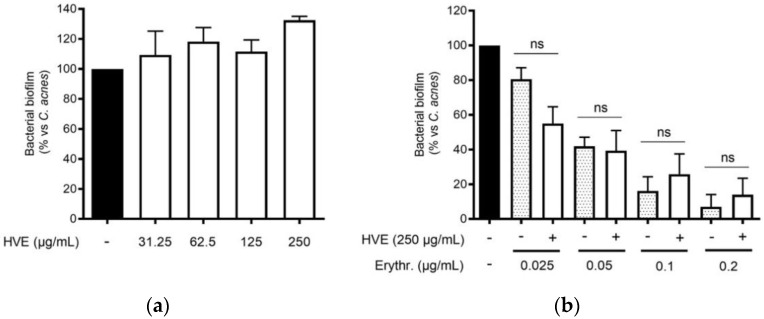
Effect of HVE treatment (125–1000 μg/mL) on the biofilm formation of *C. acnes* (O.D. = 0.1) for 24 h. Erythromycin (0.2 μg/mL) was used as the antibiotic control (−90% of growth vs. control). Evaluation of HVE alone (**a**) or in co-administration, at 250 μg/mL, with erythromycin (0.025–0.2 μg/mL) on bacterial biofilm formation (**b**). HVE + erythromycin not significant (ns) vs. equivalent concentration of erythromycin.

The traditional use of tannins, including HT, for antibacterial purposes, was sustained by scientific evidence regarding staphylococcal infections [22,33]. However, to our knowledge, *Hamamelis virginiana* L. or pure HT have never been investigated against *C. acnes* proliferation. Consequently, we supposed that a potential antibacterial activity would participate in the inhibitory effect on the NF-κB activity and IL-8 release. According to our experiments, HVE was not able to impair *C. acnes* proliferation up to the concentrations of 1000 μg/mL (Figure 3a). The same result was obtained by treating *C. acnes* with HT, still at concentrations higher than those present in the extract (6.25–50 μM) (Figure S3). In addition, we investigated the potential synergy of the highest concentration of HVE (250 μg/mL) with erythromycin, not showing significant effects in increasing or reducing the antibacterial activity of the antibiotic (Figure 3b). Likewise, HVE was unable to reduce the formation of *C. acnes* biofilms, either alone (Figure 4a) (HT 6.25–50 μM in Figure S4) or in co-treatment with erythromycin (Figure 4b). All these results show that neither HVE nor HT have antibacterial effects against *C. acnes*, at all the concentrations tested.

### 3.5. HVE Inhibits TNF-α-Induced Inflammation in HaCaT Cells

**Figure 5:**
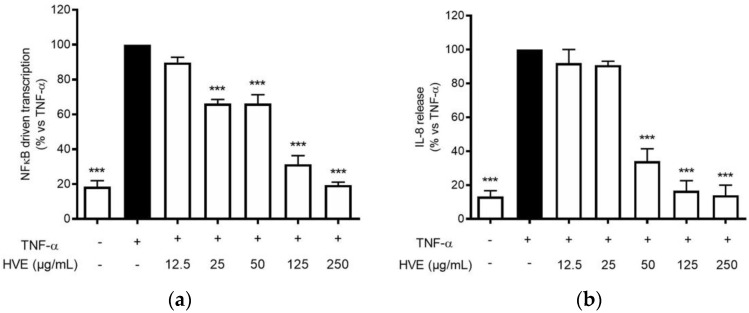
Effect of HVE treatment (12.5–250 μg/mL) on the NF-κB-driven transcription (**a**) and IL-8 release (**b**) in HaCaT cells challenged with TNF-α (10 ng/mL) for 6 h. EGCG (20 μM), representing the reference inhibitor, inhibited the NF-κB-driven transcription (−80%) and the release of IL-8 (−55%). The amount of IL-8 in the stimulated condition was 887.38 ± 39.69 pg/mL. *** *p* < 0.001 vs. stimulus.

The infection of *C. acnes* is correlated with the development of a pro-inflammatory environment after the recruitment of leukocytes in the pilosebaceous unit [34]. Typical cytokines from neutrophils, macrophages, and epithelial cells, such as TNF-α and IL-1β, are elevated in acne lesions [35]. These cytokines may in turn sustain the NF-κB activation in keratinocytes, leading to the release of IL-8 along with other mediators involved in skin lesions and fibrosis, such as metalloproteases and VEGF [36]. HVE impaired NF-κB activity in HaCaT cells during *C. acnes* infection; thus, the same mechanism was supposed to reflect in the inhibition of TNF-α-inducible mediators. Consequently, HaCaT cells were treated with TNF-α (10 ng/mL) along with HVE or HT, and the bioactivity on the NF-κB-driven transcription was evaluated. In this context, HVE showed an inhibitory effect with a lower IC_50_ (46.77 ± 6.14 μg/mL) (Figure 5a), while HT was not active up to the highest concentration tested of 5 μM (data not shown). Moreover, HVE completely abrogated the TNF-α-induced release of IL-8 at the concentrations above 125 μg/mL, reporting an IC_50_ of 38.93 ± 3.48 μg/mL (Figure 5b). In this context, it was not possible to evaluate the effect on IL-6 secretion not induced by TNF-α (10 ng/mL) until 24 h of treatment (data not shown).

Finally, both TNF-α-induced and *C. acnes*-induced nitric oxide (NO) release was evaluated in HaCaT cells in the presence of HVE. NO is known to participate in the first antibacterial response, but also to generate local oxidative stress [37]. Since NF-κB is known to induce the expression of iNOS, leading to NO production, we evaluated the effect of HVE and HT on NO release during TNF-α or *C. acnes* infection. In the first case, TNF-α was not able to induce a statistically significant release of NO at 24 h, while *C. acnes* increased the release of NO approximately four times compared to unstimulated controls, but HVE did not significantly reduce the secretion up to 250 μg/mL (Figure S5).

### 3.6. Comparison of Pro-Inflammatory and Pro-Oxidant Stimuli on the Effect of HVE

**Figure 6:**
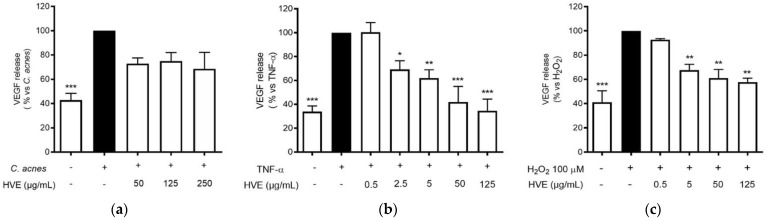
Effect of HVE treatment (0.25–25 μg/mL) on VEGF release induced by *C. acnes* (**a**), TNF-α (10 ng/mL) (**b**), or H_2_O_2_ (100 μM) (**c**) in HaCaT cells after 24 h. Apigenin (20 μM), representing the reference inhibitor, inhibited VEGF release induced by H_2_O_2_ (−57%), TNF-α (−43%), and *C. acnes* (−33%). The amount of VEGF in the presence of TNF-α or *C. acnes* was 407.48 ± 3.98 pg/mL, and 47.29 ± 2.27 pg/mL in the presence of H_2_O_2._ * *p* < 0.05, ** *p* < 0.01, *** *p* < 0.001 vs. stimulus.

The anti-inflammatory results suggested verifying the effects on another NF-κB-dependent mediator of skin inflammation and remodeling, such as VEGF [36]. VEGF-A is a key regulator of angiogenesis, recently associated with acne disease [38]. The choice to compare the effect of HVE on this parameter falls on the fact that VEGF secretion is inducible in HaCaT cells with different pro-inflammatory and pro-oxidant stimuli, as previously demonstrated [39]. All the different stimuli (*C. acnes*, TNF-α, and H_2_O_2_) induced the release of a comparable amount of VEGF compared to the unstimulated control. However, the *C. acnes*-induced VEGF secretion was not significantly reduced by HVE over the range of 50–250 μg/mL; all the concentrations reduced the secretion by about 50% without exhibiting a concentration-response mechanism (Figure 6a). The greatest inhibitory effect was obtained with the stimulation of TNF-α, in which HVE showed an IC_50_ of 15.58 ± 5.18 μg/mL (Figure 6b), about half of the IC_50_ obtained with the stimulation of H_2_O_2_ (31.12 ± 11.20 μg/mL) (Figure 6c). HT was not active in all the tested parameters (Figure S6). These results demonstrate that HVE mainly acts by blocking the inflammatory signaling pathway, only partially involving the additional antioxidant potential.

### 3.7. Contribution of Catechins to the NF-κB Inhibitory Activity of HVE

**Figure 7:**
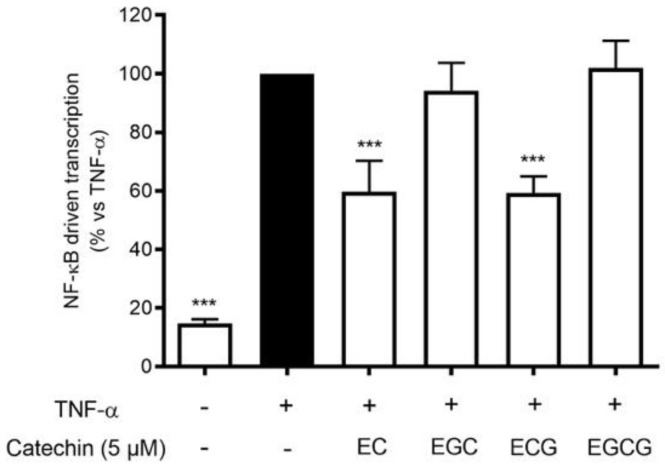
Effect of catechin treatment (5 μM) on the NF-κB-driven transcription in HaCaT cells induced by TNF-α (10 ng/mL) for 6 h. The reference inhibitor apigenin (20 μM), representing the positive control, abrogated the NF-κB-driven transcription and inhibited the release of IL-8 (−99%). The amount of IL-8 in the stimulated condition was 426.41 ± 96.97 pg/mL. *** *p* < 0.001 vs. stimulus.

Proanthocyanidins are the result of flavanol condensation, and their characteristics depend on the type of monomers and the length of the polymers of which they are constituted. The phytochemical analysis demonstrated a clear prevalence of (−)-epicatechin 3-gallate (ECG) monomers polymerized in the proanthocyanidins of HVE. For this reason, we investigated the contribution of galloylated moieties to the inhibitory activity against TNF-α-induced NF-κB activity. ECG activity was compared with (−)-epicatechin (EC), (−)-epigallocatechin (EGC), and (−)-epigallocatechin 3-gallate (EGCG), all at 5 μM concentrations (Figure 7). The results show that ECG and EC are the most active in inhibiting this parameter (Figure 7), suggesting a possible explanation of the biological activity of HVE, considering the ECG relative abundance.

## 4. Discussion

In European and North American traditional care, *Hamamelis virginiana* L. has been traditionally used in cosmetics and medicinal products for skin inflammation related to eczema, psoriasis, acne, and other skin infections. Following the traditional preparations, summarized by the HMPC monograph (EMA/HMPC/114585/2008), the market displays extracts showing non-homogeneous features (in terms of plant tissue origin or mode of extraction). For example, distillates from twigs (hamamelidis aqua), which are rich in volatile compounds but devoid of tannins, find similar applications to polar extracts from twigs and bark (hamamelidis cortex), which, contrarily, are rich in tannins. Of note, the attribution of the putative biological activity to volatile compounds or tannins still requires pharmacological validation.

The present work characterized a glycolic extract (HVE) commercially available for the presence of HT and condensed tannins. According to the literature, regarding polar extracts from twigs and bark, HT represented the most abundant individual compound in the extract (0.29% *w*/*w*); polymers belonging to the class of condensed tannins occurred in large amounts in the extract (0.30% *w*/*w*). The latter were characterized by a high degree of polymerization (aDP = 19) and a high prevalence of galloylated units, of which epicatechin gallate (ECG) was the most recurrent.

The first experiments regarding the antioxidant activity suggested that HVE may moderately prevent ROS formation in keratinocytes (HaCaT cells), mainly through direct radical scavenging. However, from the comparison among HVE and its main compound HT, the latter showed only a marginal role in the bioactivity of the extract.

HVE (5–250 μg/mL) also demonstrated inhibitory activity against the NF-κB-driven transcription and the correlated IL-6 and IL-8 release in keratinocytes challenged with *C. acnes* infection or TNF-α, suggesting a possible role against the topical inflammation occurring in acne. Moreover, the release of VEGF during TNF-α induction was also impaired, inhibiting another parameter associated with acne lesions. In this context, the participation of HT in the biological effect of HVE was excluded, reinforcing the suggestion of the fundamental role played by condensed tannins.

On the contrary, NO release was not impaired by HVE, suggesting the preservation of the first antibacterial response occurring in keratinocytes. On the other side, these data suggest a lower relevance for the antioxidant activity with respect to the anti-inflammatory activity of HVE at the skin level.

In line with this hypothesis, ECG—intended as a pharmacophore of the galloylated-proanthocyanidins—inhibited the NF-κB-driven transcription induced by TNF-α at low μM levels. Moreover, neither HVE (1000 μg/mL) nor HT (25 μg/mL) counteracted the growth of *C. acnes*, although the antibacterial effect is one of the main activities attributed to *Hamamelis virginiana* L., previously demonstrated versus staphylococcal and streptococcal species [22]. However, this work neither allows excluding the synergistic effects of tannin components for the bioactivity nor the role of HT in other biological contexts. Although the HVE concentrations used in this study may seem high, it must be considered that the biological activities herein demonstrated occurred at concentrations of condensed tannins that were over 300 times lower in respect to those of HVE. In the future, different extraction techniques may be used to concentrate these specific components for further studies.

## 5. Conclusions

This is the first demonstration of the potential anti-inflammatory mechanisms of *Hamamelis virginiana* L. twig and bark extract in the context of acne infection. In conclusion, the best anti-inflammatory effects resulted when HaCaT cells were stimulated by TNF-α, suggesting further investigations of HVE regarding other inflammatory-based skin diseases.

## Figures and Tables

**Table 1:** Phytochemical composition of *Hamamelis virginiana* L. extract (HVE).

Class	Compounds		Amount
Phenolic acids	4-hydroxybenzoic acid	µg/g	2.68
	Vanillin	µg/g	0.11
	Vanillic acid	µg/g	0.87
	Protocatechuic acid	µg/g	0.56
	Methyl gallate	µg/g	0.07
	Ferulic acid	µg/g	0.45
	Ellagic acid	µg/g	76.00
Flavonoids	Naringenin	µg/g	0.22
	Quercetin-3-O-glucoside	µg/g	0.56
	Isorhamnetin-3-O-glucoside	µg/g	0.56
Flavan-3-ols (catechins)	Catechin (free)	µg/g	143.30
	Epicatechin (free)	µg/g	0.67
	Total flavanols monomers	µg/g	230.13
	Gallocatechin (free)	µg/g	76.44
	Epigallocatechin (free)	µg/g	0.67
Gallotannins	Hamamelitannin	µg/g	2910.21
Procyanidins dimers	Procyanidin B1	µg/g	18.08
	Procyanidin B2	µg/g	0.00
	Total procyanidins dimers	µg/g	18.08
Oligomeric and polymeric proanthocyanidins	Catechin (after Phl.)	µg/g	221.53
	Catechin (terminal units)	µg/g	78.23
	Epicatechin (after Phl.)	µg/g	13.79
	Epicatechin (terminal units)	µg/g	13.12
	Gallocatechin (after Phl.)	µg/g	127.79
	Gallocatechin (terminal units)	µg/g	51.34
	Epigallocatechin (after Phl.)	µg/g	2.46
	Epigallocatechin (terminal units)	µg/g	1.79
	Catechin Gallate +		
	Epicatechin Gallate (free)	µg/g	9.03
	Catechin Gallate +		
	Epicatechin Gallate (after Phl.)	µg/g	20.21
	Catechin Gallate +		
	Epicatechin Gallate (terminal)	µg/g	11.15
	Catechin-Phl.	µg/g	0.00
	Catechin + Epicatechin-Phl.	µg/g	298.64
	Epigallocatechin-Phl	µg/g	310.02
	Epicatechin Gallate-Phl	µg/g	2213.26
	% Galloylation (terminal units)	%	3.21
	% Galloylation (upper and extension units)	%	78.43
	Total terminal units	µg/g	155.64
	Total upper units	µg/g	2821.92
	Total polymeric proanthocyanidins	µg/g	2977.56
	aDP (average degree of polymerization)		19.1

**Table 2:** Values of oxygen radical absorbance capacity (ORAC) of HVE and HT.

ORAC Test	mmol Trolox eq./g HVE	±SEM
HVE	5.519	0.512
HT	0.389	0.002

**Table 3:** Values of DPPH radical scavenging activities of HVE and HT.

DPPH Test	µmol Trolox eq./g HVE	±SEM
HVE	90.223	0.284
HT	16.760	0.976

## Data Availability

The data presented in this study are available upon request from the corresponding author.

## Supplementary Materials

The following supporting information can be downloaded at: https://www.mdpi.com/article/10.3390/antiox11061119/s1, Figure S1: Effect of HT treatment on the NF-κB-driven transcription; Figure S2: Effect of HT treatment (6.25–50 μM) on the growth of *C. acnes* (O.D. = 0.1) for 24 h; Figure S3: Effect of HT treatment (6.25–50 μM) on the biofilm formation of *C. acnes* (O.D. = 0.1) for 24 h; Figure S4: Effect of HVE treatment (50–250 μg/mL) on the NO release (b) in HaCaT cells infected with *C. acnes* (O.D. = 0.1) for 24 h. EGCG (40 μM), representing the reference inhibitor, inhibited the release of NO (−57%); Figure S5: Effect of HT treatment (5 μM) on VEGF release induced by *C. acnes* (a), TNF-α (10 ng/mL) (b), or H2O2 (100 μM) in HaCaT cells after 24 h.
