# Supplementary material for: Anti-Inflammatory and Anti-Acne Effects of Hamamelis virginiana Bark in Human Keratinocytes
Source: Antioxidants (Basel). 2022 Jun 5;11(6):1119. doi: 10.3390/antiox11061119 (PMC9220085; doi:10.3390/antiox11061119)
Supplement: Supplementary file 1 [file antioxidants-11-01119-s001.zip › antioxidants-1713960-supplementary.pdf]

Supplementary material

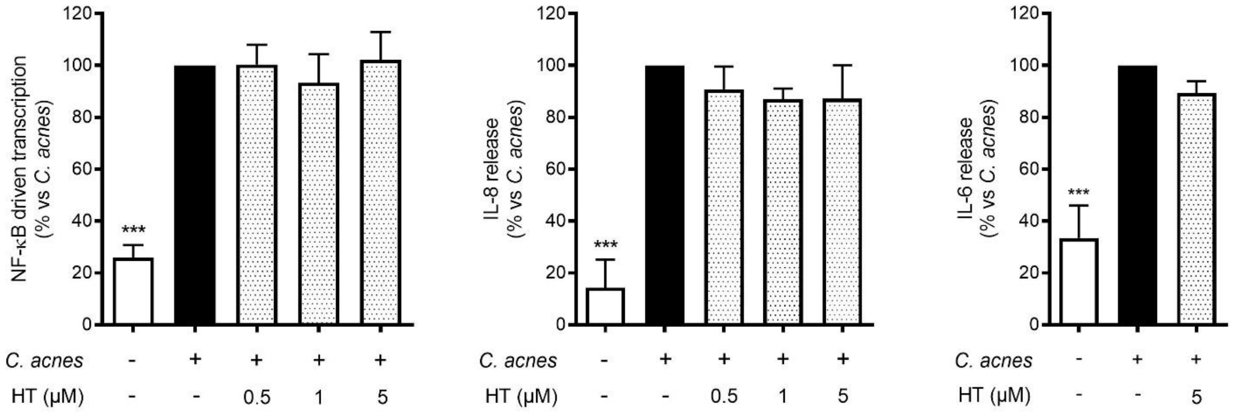

(a) (b) (c)

Figure S1. Effect of HT treatment on the NF-κB-driven transcription (0.5-5 µM) (a) for 6 h, IL-8 release (0.5-5 µM) (b), and IL-6 release (5 µM) (c) in HaCaT cells infected with *C. acnes* (O.D.=0.1) for 24 h. Apigenin (20 µM), representing the reference inhibitor, abrogated the NF-κB-driven transcription (-100%) and inhibited the release of IL-8 (-42%) and IL-6 (-97%). The amount of IL-8 in the stimulated condition was 426.41 ± 96.97 pg/mL. *** $p < 0.001$ vs. stimulus.

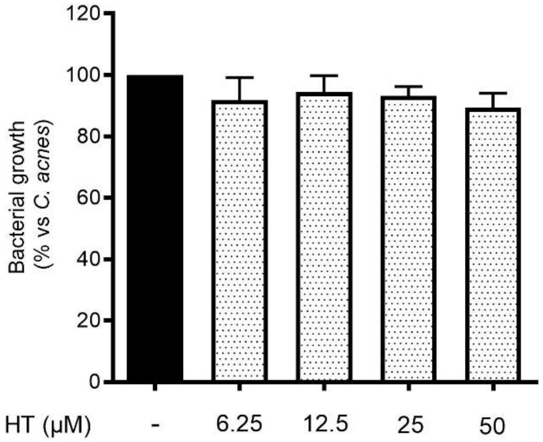

Figure S2. Effect of HT treatment (6.25-50 µM) on the growth of *C. acnes* (O.D.=0.1) for 24 h. Erythromycin (0.2 µg/mL) was used as antibiotic control (-80% of growth vs. control).

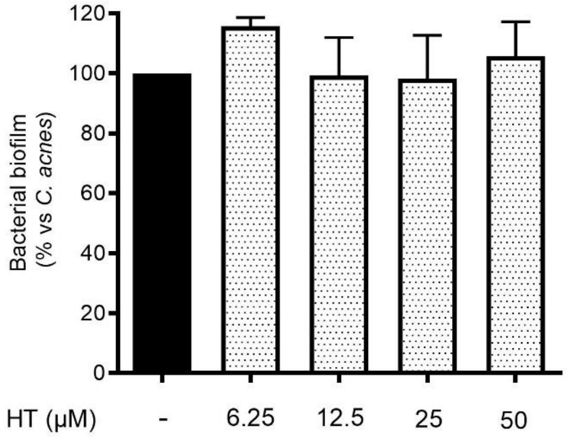

Figure S3. Effect of HT treatment (6.25-50 μM) on the biofilm formation of *C. acnes* (O.D.=0.1) for 24 h. Erythromycin (0.2 μg/mL) was used as antibiotic control (-90% of growth vs. control).

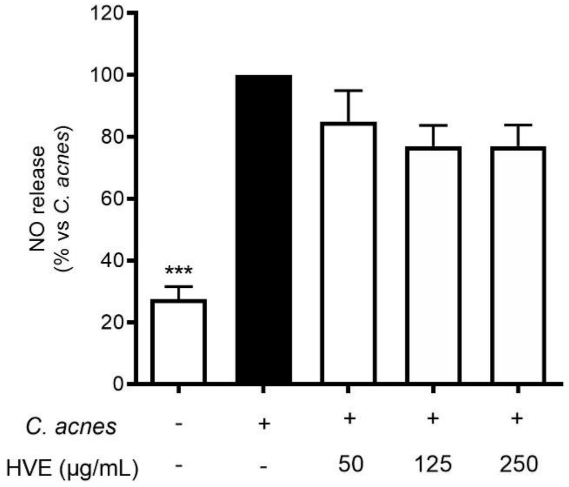

Figure S4. Effect of HVE treatment (50-250 μg/mL) on the NO release (b) in HaCaT cells infected with *C. acnes* (O.D.=0.1) for 24 h. EGCG (40 μM), representing the reference inhibitor, inhibited the release of NO (-57%). *** $p < 0.001$ vs. stimulus.

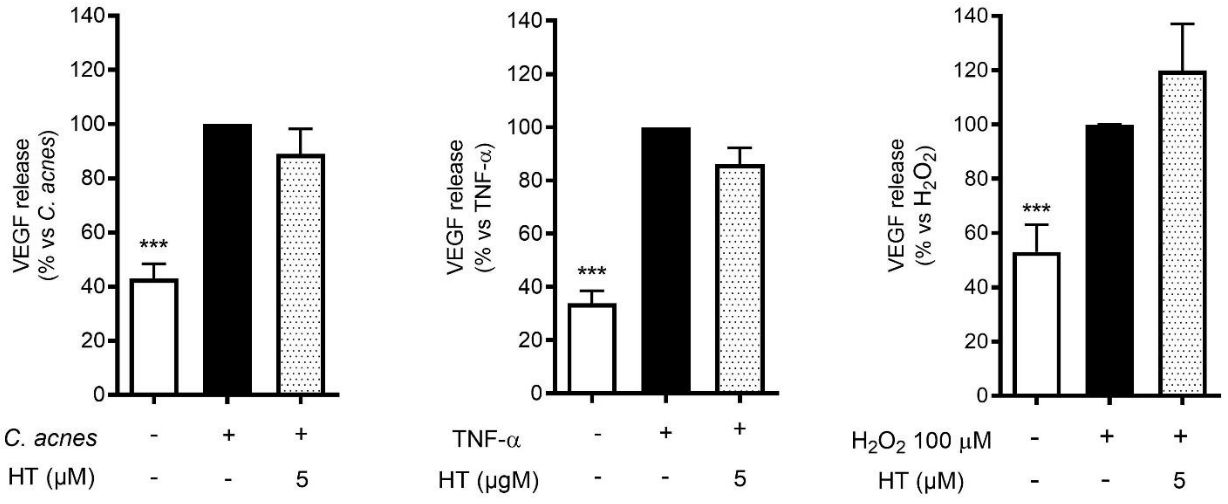

(a) (b) (c)

Figure S5. Effect of HT treatment (5 µM) on VEGF release induced by *C. acnes* (a), TNF-*α* (10 ng/mL) (b), or H_2O_2 (100 µM) in HaCaT cells after 24 h. Apigenin (20 µM), representing the reference inhibitor, inhibited VEGF release induced by H_2O_2 (-57%), TNF-*α* (-43%) and *C. acnes* (-33%). The amount of VEGF in the presence of TNF-*α* or *C. acnes* were 407,48 ± 3.98 pg/mL, and 47,29 ± 2,27 pg/mL in the presence of H2O2. *** $p < 0.001$ vs. stimulus.
